# Identification of uPAR Variants Acting as ceRNAs in Leukaemia Cells

**DOI:** 10.3390/cancers14081980

**Published:** 2022-04-14

**Authors:** Mariaevelina Alfieri, Anna Li Santi, Luigia Meo, Valentina Giudice, Carmine Selleri, Pia Ragno

**Affiliations:** 1 Department of Chemistry and Biology, University of Salerno, Via Giovanni Paolo II, 132, 84084 Salerno, Italy; malfieri@unisa.it (M.A.); alisanti@unisa.it (A.L.S.); lmeo@unisa.it (L.M.); 2 Clinical Pathology, Pausilipon Hospital, A.O.R.N Santobono-Pausilipon, 80129 Naples, Italy; 3 Department of Medicine and Surgery, University of Salerno, Via Giovanni Paolo II, 132, 84084 Salerno, Italy; vgiudice@unisa.it (V.G.); cselleri@unisa.it (C.S.)

**Keywords:** uPAR, urokinase receptor, ceRNA, AML, microRNA

## Abstract

**Simple Summary:**

The urokinase (uPA) receptor (uPAR) concentrates proteolytic activities on the cell surface and is an adhesion receptor for vitronectin. Urokinase/Vitronectin binding to uPAR activates intracellular signals promoting cell adhesion, migration, proliferation and survival. Thus, uPAR can sustain most activities of malignant cells and, accordingly, increased uPAR expression is associated with poor prognosis in several malignancies. We previously demonstrated that, in leukaemia cells, the uPAR 3′untranslated region (3′UTR) up-regulates the expression of pro-tumoral factors by recruiting microRNAs targeting their mRNAs, thus acting as competitive endogenous RNA (ceRNA). Here, we identify 3′UTR-containing variants of uPAR mRNA in leukaemia cells and demonstrate that the over-expression of uPAR Δ5-variant mRNA promotes expression of pro-tumoral factors and increase in biological activities, probably by its ceRNA activity. On this basis, we propose that uPAR may play a crucial role in cancer biology also at mRNA level, through the ceRNA activity of its variants.

**Abstract:**

The 3′untranslated region (3′UTR) of the urokinase (uPA) receptor (uPAR) mRNA can act as a competitive endogenous RNA (ceRNA) in acute myeloid leukaemia (AML) cells, promoting the expression of pro-tumoral targets, including uPAR. Here, we identified three variants of uPAR mRNA containing the 3′UTR, in KG1 and U937 leukaemia cells expressing low and high uPAR levels, respectively. Identified variants lack exon 5 (uPAR Δ5) or exon 6 (uPAR Δ6) or part of exon 6, exon 7 and part of 3′UTR (uPAR Δ6/7). uPAR Δ5 and uPAR Δ6 transcript levels were higher in U937 cells compared to KG1 cells. Both uPAR variants were expressed also in AML blasts, at higher levels as compared to CD34 hematopoietic cells from healthy donors. The presence of the 3′UTR conferred high instability to the uPAR Δ5 variant transcript, preventing its translation in protein. Overexpression of the uPAR Δ5-3′UTR variant regulated the expression of some pro-tumoral factors previously reported to be regulated by the 3′UTR of uPAR and increased KG1 cell adhesion, migration and proliferation. These results demonstrate the expression of uPAR mRNA variants containing the 3′UTR in AML cells and the ceRNA activity and the biological effects of the uPAR Δ5-3′UTR variant.

## 1. Introduction

The urokinase-plasminogen activator (uPA) receptor (uPAR) is a three-domain receptor anchored to the cell surface by a glycosyl-phosphatidylinositolic (GPI) tail, that plays a crucial role in uPAR functions [1,2]. uPA converts plasminogen into plasmin, a broad-spectrum protease, able to promote extracellular matrix (ECM) degradation. uPAR, by binding uPA, concentrates proteolytic activities on the cell surface, favouring cell migration through degraded ECM. uPAR also acts as an adhesion receptor for vitronectin (VN), a component of provisional ECM, and regulates integrin activity [1].

uPA/VN binding to uPAR or uPAR overexpression activates intracellular signalling pathways promoting cell adhesion, migration, proliferation and survival, even if uPAR lacks transmembrane and cytosolic domains; in fact, uPAR can associate with transmembrane proteins, such as integrins and the receptors for the formylated peptide fMLF (FPRs), which may be considered its signalling partners [3,4]. uPAR is involved in several physiologic and pathologic processes including inflammation [5,6], angiogenesis [7] and cancer [8]. Indeed, uPAR can sustain most of the activities of malignant cells; accordingly, increased uPAR expression is associated with poor prognosis in several malignancies [8].

uPAR expression can be regulated at transcriptional and post-transcriptional level [9,10]. Key players of post-transcriptional regulation are small non-coding RNAs (miRs), that pair to complementary sequences, termed miRNA Response Elements (MREs), generally located in the 3′untranslated region (3′UTR) of RNA messengers [11]. Dysregulation of miR expression has been described in several cancers [12] and haematological malignancies, including acute myeloid leukaemia (AML) [13,14]; thus, miR levels have rapidly emerged as valuable diagnostic markers. MiRs also represent promising targets or tools in cancer therapeutics since some miRs can act as oncogenes, down-regulating the expression of oncosuppressor genes, whereas other miRs exert oncosuppressor activities [15,16,17].

In the last decade, various studies demonstrated the crucial role that the cross-talk among various RNA species plays in the regulation of gene expression. In particular, MREs-containing RNAs can recruit specific miRs, thus up-regulating the expression of their targets. These RNAs, also termed competitive endogenous RNAs (ceRNAs), can be long non-coding RNAs (lncRNAs), circular RNAs (cRNAs), transcripts of pseudogenes as *PTENP1* and *KRAS1P*, or protein-coding mRNAs, as CXCR4 and CCR2 mRNAs [18,19,20].

LncRNAs, cRNAs or mRNAs with ceRNA activity have been reported also in AML [21,22].

We previously showed that, in leukaemia cell lines and in blasts from AML patients, uPAR mRNA can be targeted by two oncosuppressor miRs, miR-146a and miR-335. Accordingly, KG1 acute myelogenous leukaemia cells showed low uPAR expression and high levels of miR-146a and miR-335, whereas U937 pro-monocytic leukaemia cells showed high uPAR expression and low levels of same miRs [23]. Moreover, we demonstrated that, in KG1 cells, the 3′UTR of uPAR up-regulates the expression of uPAR and other pro-tumoral factors by recruiting miRs targeting their mRNAs. Interestingly, U937 cells showed additional uPAR transcripts containing the 3′UTR, beside the full-length uPAR mRNA, as compared to KG1 cells [24]. We then hypothesized that these transcripts, differentially expressed in U937 cells, may act as ceRNAs.

On these bases, we here aimed to identify and characterize variants of uPAR transcripts carrying the 3′UTR, thus potentially being able to act as miR sponge in AML cell lines and blasts. Furthermore, we aimed to investigate their levels, functions and possible impact on AML cell biology.

## 2. Materials and Methods

### 2.1. Reagents

The anti-uPAR monoclonal antibody (mAb), clone R4 (MON R-4-02), used for Western blot analysis, was from Thermo Fisher Scientific (Hanover Park, IL, USA); the anti-uPAR polyclonal antibody (sc-10815) used in cell migration assays, and anti-Myc (sc-40), anti-ICAM-1 (sc-8439) and anti-Cathepsin (sc-376803) antibodies, used for Western blot analysis, were purchased from Santa Cruz Biotechnology (Santa Cruz, CA, USA); the anti-Enolase antibody (E-AB-31325) was from Elabscience (Houston, TX, USA).

Anti-tubulin (T5168) and anti-TfR1 (MABS1982) mAbs, the protease inhibitor cocktail and Collagen VI were from Sigma-Aldrich (Saint Louis, MO, USA). Anti-GAPDH antibodies (mAb G041 and rabbit polyclonal Y058203) were from abm (Vancouver, BC, Canada). Lymphoprep was from Stem cell Technologies (Vancouver, BC, Canada); anti-CD3 Abs and IgG-conjugated magnetic beads for immunodepletion were from Life Technologies (Carlsbad, CA, USA). The Nucleofector kit was from Lonza (Basel, Switzerland). Horseradish peroxidase-conjugated anti-mouse and anti-rabbit IgG, the protein colorimetric assay and IQ™SYBR Green Supermix were from Bio-Rad (Hercules, CA, USA). The ECL detection kit was from Amersham International (Amersham, UK) and polyvinylidene fluoride (PVDF) membrane from Millipore (Burlington, MA, USA). The chemotaxis polyvinylpyrrolidone-free (PVPF) filters from Whatman Int. (Kent, UK). QuantiTect Reverse Transcription kit, QIAquick PCR purification kit and QIAzol reagent were from Qiagen (Hilden, Germany). RNase-free DNAse I, the Superscript Reverse Transcriptase III, the Taq polymerase and the Platinum Superfi DNA polymerase were from Invitrogen (Carlsbad, CA, USA).

### 2.2. Cell Culture

The KG1 acute myelogenous leukaemia cell line was cultured in IMDM (Gibco, Thermo Fisher Scientific, Waltham, MA, USA) supplemented with 20% heat-inactivated foetal bovine serum (FBS) (Euroclone, Pero, Italy). The U937 pro-monocytic leukaemia cell line was cultured in RPMI 1640 (Gibco, Thermo Fisher Scientific, Waltham, MA, USA) supplemented with 10% heat-inactivated FBS. Both cell lines were from ECACC (Sigma-Aldrich, Saint Louis, MO, USA).

### 2.3. Patient Specimen Collection

Heparinized bone marrow (BM) samples were obtained with informed consent, in accordance with protocols approved by the Ethics Committee (“Campania Sud”, ASL Napoli 3 Sud, Naples, Italy; prot./SCCE n. 24988). A total of 20 newly diagnosed AML patients were enrolled and BM mononuclear cells (BMMCs) were isolated by Ficoll–Paque density gradient centrifugation using Lymphoprep. Samples were enriched using antibodies and magnetic beads, as previously described [25], resulting in a final blast purity ≥ 95% as determined by morphology on cytospin preparations.

### 2.4. RT-PCR

Cells were lysed in QIAzol and total RNA was isolated according to the manufacturer’s instructions. For semi-quantitative RT-PCR, 1 μg of total RNA, previously treated with RNase-free DNAse I, was reverse-transcribed using random hexamers and the Superscript III RT at 50 °C for 50 min. 1 μL of reverse-transcribed DNA was amplified for 30 cycles with 2.5 units of Taq polymerase using specific primers and analysed in agarose gel.

For quantitative RT-PCR (qRT-PCR), 1 μg of total RNA was reverse-transcribed and 1 μL of a 1:10 dilution was analysed by qRT-PCR with a BioRad IQ5 system, using IQTM SYBR Green Supermix for qPCR kit, according to the manufacturer’s instructions. mRNA levels were normalized to the glyceraldehyde-3-phosphate dehydrogenase (GAPDH) mRNA levels. The relative levels of expression were calculated with the formula 2^−ΔΔ*CT*^.

Primers, designed using Primer3 software, were as follows: for uPAR amplification, uPAR forward (fw) primer 5′-CTGGAGCTGGTGGAGAAAAG-3′ and reverse primer uPAR 5′-CATGTCTGATGAGCCACAGG-3′; for uPAR Δ5 amplification, fw primer 5′-GGAAGGTGAAGAAGTCCTGG AGCT-3′ (overlapping exons 4/6 splice site) and reverse primer 5′-ATATAGCTTTGGTTTTTCGGTTCGT-3′; for uPAR Δ6 amplification, fw primer 5′-TGCAACGAGGGCCCAAACCGAA-3′ (overlapping exons 5/7 splice site) and reverse primer 5′-CAGGTCTGGGTGGTTACAGC-3′; for uPAR Δ6/7 amplification fw primer 5′-GCTCCAATGGTTTCCACAACAACGA-3′ and reverse primer 5′-CATAGCTGGGAAAACTAAGGAAAGTC-3′(overlapping the exon 6/3′UTR splice site); for GAPDH amplification, fw primer 5′ GAAGGTGAAGGTCGGAGT-3′ and reverse primer 5′-GAAGATGGTGATGGGATTT-3′.

### 2.5. Identification of uPAR Transcript Variants

Total RNA from KG1 and U937 cells was reverse-transcribed and used for five PCRs with uPAR specific primers (ATG fw primer 5′-ATGG GTCACCCGCC GCTG-3′ and 3′UTR reverse primer 5′-CCACTGGTACAAAATCTTTATG-3′). PCR products were cloned into the pCR2.1 vector of the TOPO-TA Cloning system (Termofisher Scientific, Il, USA); the cloning reaction was used to transform One Shot™ TOP10 Chemically Competent *E. coli* (Invitrogen, Waltham, MA, USA) cells, according to manufacturer’s instructions. A total of 100 colonies were selected and analysed for the insert by colony PCR with M13 primers (fw primer 5′-GTAAAACGACGGCCAG-3′ and reverse primer 5′-CAGGAAACAGCTATGAC-3′). Plasmid DNA was extracted from positive colonies and sequenced (BMR Genomics, Padova, Italy).

### 2.6. Cloning of 3′UTR-Containing Full Length uPAR and uPAR Δ5 Variant

Total RNA isolated from U937 cells was reverse-transcribed and uPAR, from its ATG to the 3′UTR (for full length uPAR and uPAR Δ5 variant carrying the 3′UTR) or to the stop codon (for uPAR Δ5 variant lacking the 3′UTR), was amplified by PCR using the Platinum Superfi DNA polymerase and specific primers. Primers were designed to allow cDNA cloning in KpnI and EcoRI sites of the pcDNA3 expression vector (KpnI-uPAR/ATG fw primer 5′-**CGGGGTACC**ATGGGTCACCC GCCGCTGCTGCCG-3′ and EcoRI-3′UTR reverse primer 5′-**CCGGAATTC**CCACTGGTACAAAA TCTTTATGTAAGT-3′ or EcoRI-stop codon reverse primer 5′-**CCGGAATTC**TTAGGTCCAGAGGAGAGTGCCTCCCCA-3′). PCR products were loaded onto agarose gel and the bands corresponding to full length uPAR and uPAR Δ5 (with or without the 3′UTR) were excised, the DNA was purified by a QIAquick PCR purification kit. The constructs were cloned into the KpnI and EcoRI sites of pcDNA3 vector and checked by sequence analysis (BMR Genomics, Padova, Italy).

### 2.7. Transfections

KG1 cells were transfected by electroporation using Amaxa™ Nucleofector™ Technology, according to the protocol specifically indicated by the manufacturer (Lonza, Switzerland) and previously described [24]. A total of 2 × 10^6^ cells were transfected with 2 µg of DNA in 100 µL of HBSS buffer and then diluted to 1.6 mL and incubated for the indicated times or cultured for 15 days in selection medium added with 0.4 mg/mL G418 to obtain stably transfected cells.

### 2.8. Western Blot Analysis

Cells were lysed in 1% Triton X-100 and the protein content measured by a colorimetric assay. Cell lysates were electrophoresed in SDS-PAGE, transferred onto a PVDF filter, blocked with 5% milk and probed with primary antibodies. Washed filters were incubated with horseradish peroxidase-conjugated secondary antibodies and bands detected by ECL.

### 2.9. Cell Migration Assay

A total of 2 × 10^5^ cells, resuspended in serum-free medium, were loaded onto uncoated PVPF polycarbonate filters (5 µm pore size) in the upper compartment of Boyden chambers; medium supplemented with 10% FBS was added in the lower compartment as chemoattractant. Cells were incubated for 2 h at 37 °C, 5% CO_2_. Then, the cells on the lower surface of the filter were fixed in 70, 90, 100% ethanol, stained with Mayer’s hematoxylin, and counted at 20× magnification (10 random fields/filter).

### 2.10. Cell Adhesion Assay

A total of 10 µg/mL of fibronectin or 1% heat-denatured BSA-PBS, as a negative control, were loaded onto flat-bottoms of 96-well microtiter plates and incubated 16 h at 4 °C. Coated plates were then incubated for 1h at room temperature with 1% heat-denatured BSA-PBS, for blocking non-specific binding sites. An amount of 100 µL of a cell suspension containing 1.5 × 10^6^ cells/mL were loaded in each coated well and incubated for 2 h at 37 °C. After washings, attached cells were fixed with 3% paraformaldehyde (PFA) at 37 °C in PBS and, then, with 20% methanol. Fixed cells were stained with 0.5% crystal violet in 20% methanol. Stain was eluted by 0.1 M sodium citrate in 50% ethanol, pH 4.2, and the absorbance at 540 nm was measured with a spectrophotometer.

### 2.11. Cell Proliferation Assay

Cells were incubated in serum-free medium for 16 h for serum starvation and then cultured with 5% FBS in IMDM; after 0, 24, 48 or 72 h, 10 µL of cell suspension was diluted in 400 µL and divided into four wells of 96-well plates. Then, 20 µL of CellTiter 96 AQueous One Solution Reagent were loaded onto each well and incubated for 4 h at 37 °C, 5% CO_2_. The absorbance was determined by an ELISA reader (BioRad, Hercules, CA, USA) at a wavelength of 490 nm.

### 2.12. Statistical Analysis

Differences between groups were evaluated by the Student’s *t*-test using PRISM software (GraphPad, San Diego, CA, USA). *p <* 0.05 was considered statistically significant.

## 3. Results

### 3.1. Identification of uPAR Variants in U937 AML Cell Line

**Figure 1:**
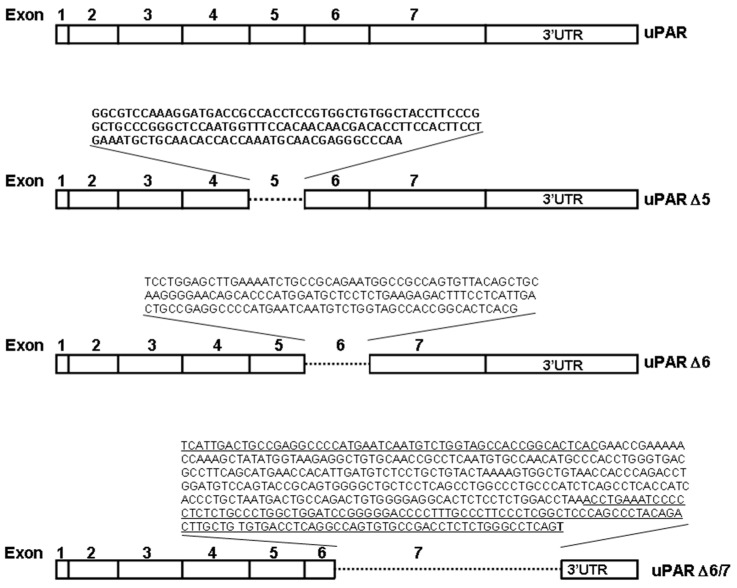
Schematic representation of identified variants of uPAR mRNA. uPAR corresponds to full length uPAR, uPAR Δ5 lacks nucleotides (nt) 473–607, uPAR Δ6 lacks nt 608–754, uPAR Δ6/7 lacks nt 701–1131 (nt were numbered from the ATG).

We aimed to identify and characterize 3′UTR-carrying uPAR transcript variants, which may act as ceRNAs in AML cells. To this end, PCRs were performed using specific primers to amplify uPAR (from its ATG to the whole 3′UTR) and, as a template, the DNA obtained by reverse-transcription of U937 total RNA. Then, PCR products were cloned in the pCR2.1 vector; 100 colonies resulting from the transformation were examined by PCR, using M13 primers, and sequenced. The sequencing revealed the presence of the cDNA of uPAR lacking exon 5 (uPAR Δ5) in 10/100 clones, of uPAR lacking exon 6 (uPAR Δ6) in 6/100 clones and of a variant lacking half exon 6, the entire exon 7 and half 3′UTR (uPAR Δ6/7) in 2/100 clones (Figure 1).

### 3.2. uPAR Variants Are Differently Expressed in AML Cell Lines

The expression of transcript variants was evaluated in U937 and KG1 cells by qRT-PCR analysis, using primers overlapping the splice site of exons 4 and 6 for uPAR Δ5 detection, of exons 5 and 7 for uPAR Δ6 detection, of exon 6/3′UTR for uPAR Δ6/7 detection. Total uPAR expression was also evaluated as a control.

**Figure 2:**
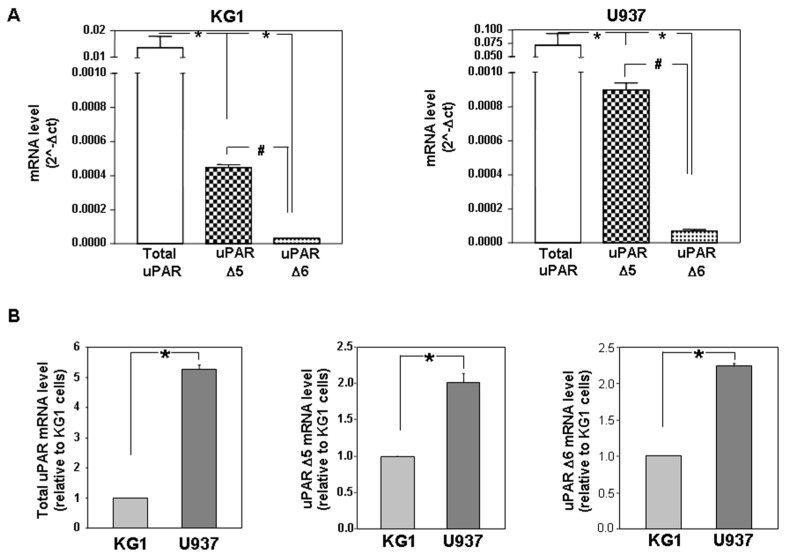
AML cell lines express different amounts of uPAR variants. (**A**) The levels of uPAR, uPAR Δ5 and uPAR Δ6 transcripts, normalized to the internal glyceraldeyde-3-phosphate dehydrogenase (GAPDH) mRNA, were analysed by qRT-PCR in KG1 (left panel) and in U937 (right panel) cells. The absolute level of expression was calculated with the formula 2^−Δ*CT*^. Values are the mean ± SEM of three experiments performed in triplicate; * *p* < 0.05 as determined by the Student’s *t* test, total uPAR vs. each variant; # *p* < 0.05 as determined by the Student’s *t* test, uPAR Δ5 vs. uPAR Δ6. (**B**) The levels of uPAR, uPAR Δ5 and uPAR Δ6 transcripts were analysed by qRT-PCR and expressed as fold change of uPAR expression in U937 cells relative to KG1 cells. The relative level of expression was calculated with the formula 2^−ΔΔ*CT*^. Values are the mean ± SEM of three experiments performed in triplicate; * *p* < 0.05 as determined by the Student’s *t* test.

U937 and KG1 cells expressed uPAR Δ5 and uPAR Δ6 variants, with uPAR Δ5 being the more abundant variant; however, the levels of both variants were very low as compared to the total uPAR levels (Figure 2A). The uPAR Δ6/7 variant was not detected probably because of its low level, accordingly with cloning results (see previous paragraph).

The levels of uPAR Δ5 and uPAR Δ6 variants, as the level of total uPAR, were significantly higher in U937 cells as compared to KG1 cells (Figure 2B).

Together, these results demonstrate that both analysed AML cell lines express uPAR Δ5 and uPAR Δ6 variants; however, the variant levels are higher in U937 cells as compared to KG1 cells.

### 3.3. 3′UTR Confers Instability to the uPAR Δ5 Transcript

uPAR transcript variants, identified in AML, could act as ceRNAs if their 3′UTR recruits miRs and regulates the expression of other targets; consequently, as a proof-of-concept, increased degradation of uPAR variant transcripts and, at the same time, increased expression of those targets should occur. We focused on uPAR Δ5 transcript, the most abundant uPAR variant in AML cells, to elucidate this aspect.

**Figure 3:**
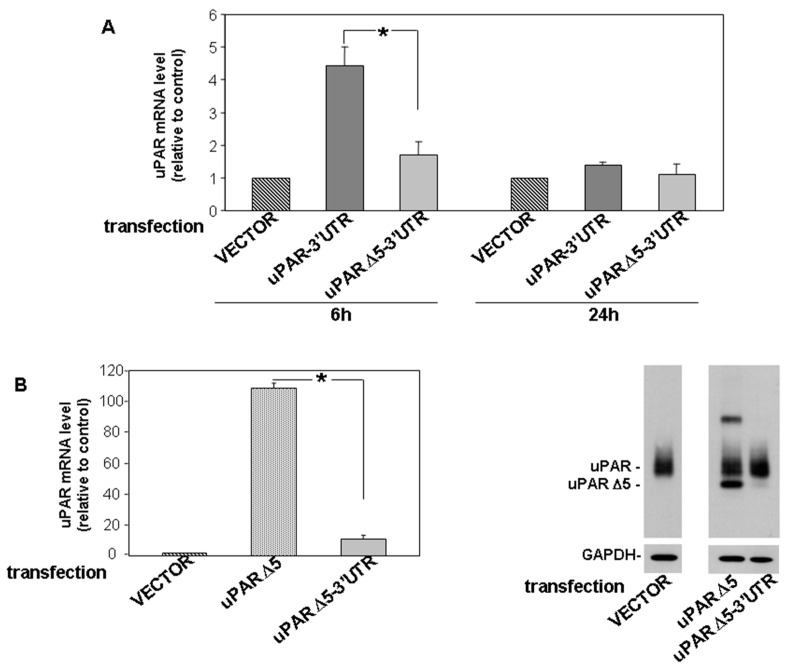
The 3′UTR confers instability to the uPAR Δ5 transcript. (**A**) KG1 cells were transiently transfected with the cDNA of uPAR-3′UTR or uPAR Δ5-3′UTR, or with the empty vector as a control; cells were harvested 6 h and 24 h after transfection. uPAR transcript levels, analysed by qRT-PCR and normalized to the GAPDH mRNA, are expressed as fold change of endogenous uPAR expressed by KG1 cells transfected with the empty vector. The relative level of expression was calculated with the formula 2^−ΔΔ*CT*^. Values are the mean ± SEM of three experiments performed in triplicate; * *p* < 0.05 as determined by the Student’s *t* test. (**B**) KG1 cells were transiently transfected with the cDNA of uPAR Δ5 carrying (uPAR Δ5-3′UTR) or not (uPAR Δ5) the 3′UTR, or with the empty vector, as a control; cells were harvested 6 h after transfection. uPAR transcript levels, analysed by qRT-PCR and normalized to the GAPDH mRNA, are calculated with the formula 2^−ΔΔ*CT*^ relative to the endogenous uPAR expressed by KG1 cells transfected with the empty vector. Values are the mean ± SEM of three experiments performed in triplicate; * *p* < 0.05 as determined by the Student’s *t*-test (left panel). Part of KG1 transfected cells were lysed in 1% TRITON X-100 and 10 µg of cell lysates were analysed by Western blot with an anti-uPAR specific antibody; the filter was then reprobed with a rabbit anti-GAPDH antibody as a loading control (right panel). Original Western Blot can be found at Supplementary Materials Figure S1.

First, we compared the degradation rate of uPAR Δ5 transcript with that of full length uPAR. The cDNA of full length uPAR and of uPAR Δ5, both containing the 3′UTR (uPAR-3′UTR and uPAR Δ5-3′UTR, respectively), were cloned in an expression vector; then, the constructs or the empty vector (negative control) were transfected in KG1 cells, expressing high levels of uPAR-targeting miRs [23]. qRT-PCR analysis of transfected KG1 cells showed expression of both uPAR transcripts 6h after transfection; however, the mRNA level of transfected uPAR Δ5-3′UTR was significantly lower than that of transfected full length uPAR-3′UTR, suggesting its faster degradation. This difference was nearly lost 24 h after transfection (Figure 3A).

Next, we investigated whether the observed instability could be caused by the 3′UTR. KG1 cells were transfected with the cDNA of uPAR Δ5 containing the 3′UTR (uPAR Δ5-3′UTR) or lacking this regulatory region (uPAR Δ5). qRT-PCR analysis of 6h transfected KG1 cells showed a very low level of uPAR Δ5-3′UTR as compared to the expression of uPAR Δ5, suggesting that the presence of the 3′UTR confers high instability to the uPAR Δ5 variant. Accordingly, this uPAR variant, at protein level, was detectable only in KG1 cells transfected with the uPAR Δ5 cDNA lacking the 3′UTR (Figure 3B).

These results suggest a faster degradation of the uPAR Δ5 transcript carrying the 3′UTR, according with the hypothesis that it acts as a miRNA sponge, while the transcript lacking the 3′UTR is more stable and can be efficiently translated in protein.

### 3.4. uPAR Δ5 Variant Regulates the Expression of Pro-Tumoral Factors

**Figure 4:**
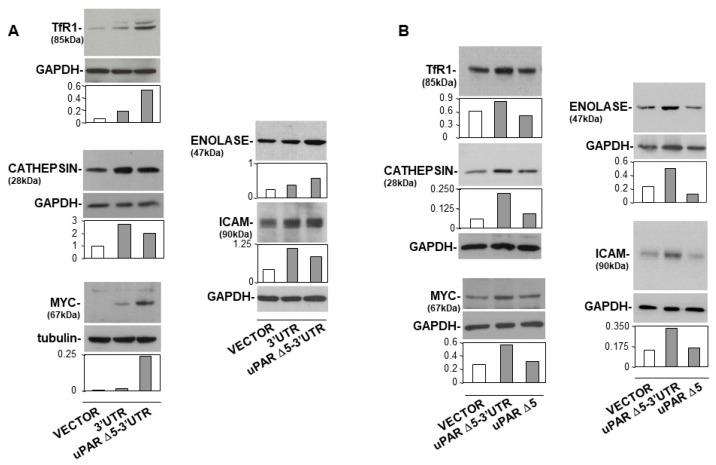
uPAR Δ5 variant regulates the expression of pro-tumoral factors. (**A**) KG1 cells were transfected with uPAR Δ5-3′UTR or with the empty vector or the 3′UTR of uPAR, as a negative and positive control, respectively; after 24 h cells were lysed. An amount of 20 µg of cell lysates was analysed by Western blot with MYC, TfR1, Enolase, Cathepsin specific antibodies. The filter hybridized with the anti-Enolase antibody was re-hybridized with an anti-ICAM antibody. All filters were then re-probed with rabbit anti-GAPDH or mouse anti-tubulin antibodies as a loading control. Detected bands were analysed by densitometric scanning and the O.D. corresponding to specific bands were normalized to the O.D. of corresponding GAPDH or tubulin bands. (**B**) KG1 cells were transfected with the cDNA of uPAR Δ5 carrying (uPAR Δ5-3′UTR) or not (uPAR Δ5) the 3′UTR, or with the empty vector. An amount of 20 µg of cell lysates was analysed by Western blot with MYC, Cathepsin, Enolase and ICAM specific antibodies; the filter hybridized with the anti-Cathepsin antibody was re-hybridized with the anti-TfR1 antibody. All filters were then re-probed with a rabbit anti-GAPDH antibody as loading control. Detected bands were analysed by densitometric scanning and the O.D. corresponding to specific bands were normalized to the O.D. of corresponding GAPDH bands. Original Western Blots can be found at Supplementary Materials Figures S2 and S3.

We previously demonstrated the ceRNA activity of the 3′UTR of uPAR and showed that its transfection in KG1 cells increased the expression of various pro-tumoral factors [24]. Here, we investigate whether the uPAR Δ5 variant of uPAR carrying the regulatory 3′UTR could exert the same ceRNA activity. First, KG1 cells were transfected with the uPAR Δ5-3′UTR construct or the empty vector or the sole 3′UTR of uPAR, as negative and positive controls, respectively. Cell lysates were then analysed by Western blot with antibodies specific for some pro-tumoral factors previously shown to be regulated by the 3′UTR of uPAR [24]. Western blot analysis showed increased expression of the transcriptional factor Myc, the transferrin receptor (TfR-1), the glycolysis-involved enolase enzyme (also known as phosphopyruvate hydratase) and the intercellular adhesion molecule-1 (ICAM-1), in KG1 cells transfected with uPAR Δ5-3′UTR or with the 3′UTR of uPAR, as compared to negative control cells transfected with the empty vector (Figure 4A).

In order to elucidate whether observed increases were due to the 3′UTR of the uPAR Δ5 variant, i.e., to its ceRNA activity, KG1 cells were transfected with the cDNA of uPAR Δ5 with or without the 3′UTR, or with the empty vector as a negative control. Western Blot analysis confirmed that only the uPAR Δ5-3′UTR increased the levels of examined pro-tumoral factors, whereas the same variant, lacking the 3′UTR, did not exert any effect on their expression (Figure 4B).

These results supported the hypothesis that the uPAR Δ5 variant has a ceRNA activity due to its 3′UTR.

### 3.5. uPAR Δ5 Variant Influences Cell Activities

We previously showed that the 3′UTR of uPAR regulates cell adhesion and migration through its ceRNA activity, whereas it does not influence cell proliferation. Based on these findings, we explored the ability of uPAR Δ5-3′UTR to influence these activities in KG1 cells stably transfected with the variant or with the 3′UTR or the empty vector as positive and negative controls, respectively.

**Figure 5:**
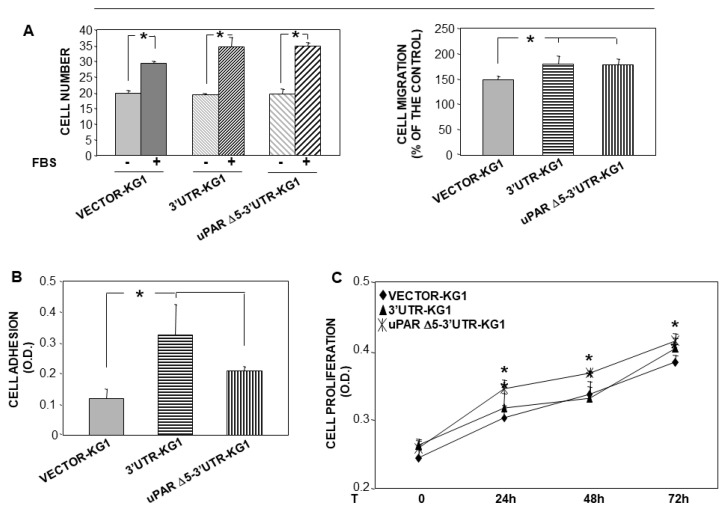
uPAR Δ5 variant influences cell adhesion, migration and proliferation. (**A**) KG1 cells were stably transfected with the cDNAs of uPAR Δ5 or of 3′UTR of uPAR, or with the empty vector, as control. A total of 2 × 10^5^ transfected cells were loaded in Boyden chambers and allowed to migrate towards 10% serum (FBS). Migrated cells were fixed, stained with haematoxylin and counted (left panel). Results of same migration assays are also expressed as percentage of cells migrated towards the chemoattractant over the cells migrated without chemoattractant; 100% value represents cell migration in the absence of chemoattractant. Values are the mean ± SEM of three experiments performed in triplicate; * *p* < 0.05, as determined by the Student’s *t*-test. (**B**) KG1 cells were stably transfected with the cDNA of uPAR Δ5 or of uPAR 3′ UTR, or with the empty vector, as control. A total of 1.5 × 10^5^ transfected cells were plated in wells pre-coated with 10 µg/mL of fibronectin (FN) or 1% BSA-PBS as a negative adhesion control, and incubated for 2 h at 37 °C, 5% CO_2_. Attached cells were fixed with 3% PFA and stained with crystal violet; the stain was eluted and its absorbance at 540 nm was measured with a spectrophotometer. Values corresponding to cells plated on FN were subtracted of values corresponding to cells plated on BSA. The values are the mean ± SEM of six experiments performed in triplicate; * *p* < 0.05, as determined by the Student’s *t*-test. (**C**) KG1 cells, stably transfected with the cDNA of uPAR Δ5 or of uPAR 3′ UTR or with the empty vector, as a control, were serum-starved for 16 h and cultured with 5% FBS in IMDM; at 0 h, 24 h, 48 h or 72 h, 20 µL of CellTiter 96 AQueous One Solution Reagent was added to each well and incubated for 4h at 37 °C, 5% CO_2_. The absorbance was determined by an ELISA reader (λ 490 nm). Values are the mean ± SEM of three experiments performed in quadruplicate; * *p* < 0.05, as determined by the Student’s *t*-test, indicates statistically significant difference compared to control cells at each indicated time.

uPAR Δ5-3′UTR-transfected KG1 cells and 3′UTR-transfected KG1 control cells showed a significant increase in cell migration compared to negative control cells (Figure 5A).

Similarly, uPAR Δ5-3′UTR transfected cells also adhered more efficiently to fibronectin (FN), a component largely present in the bone marrow stroma, as well as 3′UTR-transfected control cells, compared to negative control cells (Figure 5B).

Unexpectedly, uPAR Δ5-3′UTR expression weakly but significantly stimulated cell proliferation, in contrast to 3′UTR-transfected cells, which showed the same proliferation rate as negative control cells, as previously reported [24] (Figure 5C).

These results indicate that the expression of the uPAR Δ5-3′UTR variant positively influences examined biological activities of AML cells.

### 3.6. Expression of uPAR Variants in Leukaemia Blasts

We finally investigated the expression of uPAR variants, in particular uPAR Δ5 and uPAR Δ6, in blasts obtained from 20 AML patients and in CD34^+^ hematopoietic stem cells (HSCs) obtained from 3 healthy donors.

**Figure 6:**
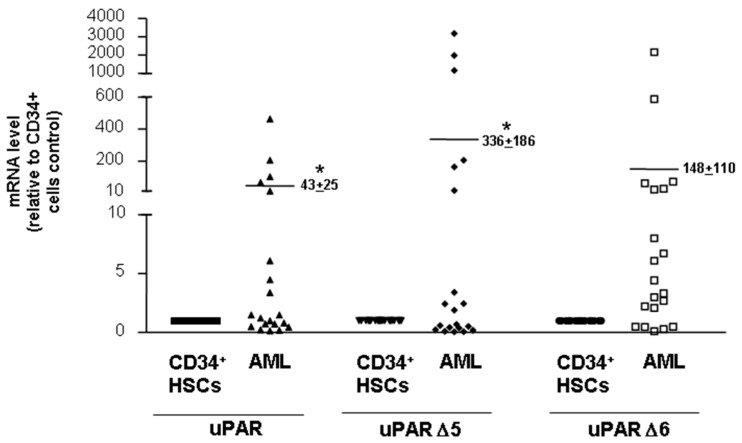
Expression of uPAR variants in leukaemia blasts. CD34^+^ hematopoietic stem cells (HSCs) from 3 healthy donors and blasts from 20 patients affected by acute myeloid leukaemia (AML) were lysed in QIAzol and analysed by qRT-PCR with uPAR, uPAR Δ5 or uPAR Δ6 specific primers. Results are expressed as fold change of uPAR expression in AML blasts relative to CD34^+^ HSCs. The relative level of expression was calculated with the formula 2^−ΔΔ*CT*^. Values are the mean ± SEM of three experiments performed in triplicate; * *p* < 0.05 as determined by Student’s *t*-test.

qRT-PCR analysis of uPAR transcripts showed a detectable expression of both variants; the mean of their level was higher in leukaemic blasts as compared to normal CD34^+^ HSCs, even though transcripts levels in AML samples were very heterogeneous (Figure 6). Observed heterogeneity in uPAR expression at mRNA level is coherent with previous reports showing heterogenous uPAR expression at protein level in blasts of AML patients [26].

These results demonstrate that uPAR variants detected in AML cell lines are expressed also in vivo in AML blasts.

## 4. Discussion

uPAR upregulation is frequently described in several solid and hematological malignancies and is associated with poor prognosis. In fact, uPAR plays a crucial role in the regulation of pericellular proteolysis and in various cell activities, supporting most cancer hallmarks [8]. We recently proposed that uPAR may play a crucial role in cancer biology also at mRNA level, since we demonstrated the ceRNA activity of the 3′UTR of uPAR mRNA, which can regulate the expression of pro-tumoral factors, including uPAR itself [24]. Based on these observations, we hypothesized that uPAR variants containing the 3′UTR regulatory region may act as ceRNAs by promoting uPAR and pro-tumoral factor expression.

uPAR gene consists of 7 exons spread over 23 kb of genomic DNA. Exon 1 includes an untranslated 5′ segment and encodes for the hydrophobic signal peptide; exons 2–3, 4–5, and 6–7 encode for uPAR DI, DII and DIII, respectively [27]. The first alternatively spliced uPAR variant was identified in various cell lines, including U937 AML cells; this variant encoded a uPAR lacking the carboxy-terminal attachment for the glycolipid anchor, leading to the soluble form of the full length uPAR [28].

The first uPAR variant lacking an entire exon was instead identified in HeLa cells, in which a novel 1.7 kb uPAR cDNA, missing exon 5 and containing 380 bp not previously reported at the 5′ end, was isolated [27].

After several years, a novel uPAR splice variant, lacking exons 4–5 (del 4/5) was identified in breast cancer cells and reported to have prognostic relevance. In fact, higher uPAR del 4/5 mRNA levels were associated with shorter disease-free survival in breast cancer patients [29] and with advanced tumour stage in soft-tissue sarcoma patients [30]; by contrast, mRNA expression level of uPAR del 4/5 was not clinically relevant in advanced ovarian cancer [31]. Unexpectedly, the protein uPAR del 4/5 led to reduced invasion through Matrigel in vitro and impaired metastatic dissemination and growth in vivo of breast cancer cells [32].

A study aimed to identify splice variants of uPAR in human cell lines and tissues (including lung, bronchial epithelium, airway smooth muscle and peripheral blood cells) was conducted in 2009 [33]. Seven major splice variants, identified by Rapid Amplification of cDNA Ends (RACE), showed exon deletions and/or an alternative exon 7b, encoding a uPAR lacking the carboxy-terminal attachment for the glycolipid anchor (causing the expression of soluble forms of uPAR). Classical exon 7 (leading to the GPI-uPAR) was preferentially expressed in peripheral blood cells, often associated with deletion of exon 6 or exons 5 + 6. All identified variants still contained the 3′UTR, which was reduced in length only in variants carrying a shorter 7b exon [33].

In the present work, we identify three variants of uPAR mRNA, lacking exon 5 (uPAR Δ5), exon 6 (uPAR Δ6) or lacking a large region encompassing half exon 6, the entire exon 7 and half 3′UTR (uPAR Δ6/7); uPAR Δ5 was previously shown in HeLa cells [27] and high levels of uPAR Δ6 have been shown in peripheral blood mononuclear cells [33], whereas uPAR Δ6/7 has never been identified. Based on previously described ceRNA activity of the 3′UTR of uPAR mRNA, we hypothesized that these variants might have a ceRNA activity due to their 3′UTR region. The level of their expression represents only a small fraction of total uPAR mRNA, but this is coherent with a possible ceRNA activity: if they act as molecular sponge for miRs they should be destined to degradation rather than translation. Indeed, uPAR Δ5 mRNA seems to degrade faster than the mRNA of full length uPAR. In this context, the less abundant variant, uPAR Δ6/7, may be the more efficient ceRNA among identified variants, even if we cannot exclude a conformational instability.

The majority of transcribed human genes can be alternatively spliced; however, alternative splicing must be tightly regulated and controlled because, if deregulated, may highly contribute to various disease, including cancer [34]. A significant deregulation of alternative splicing has been reported also in AML, with approximately one-third of expressed genes being abnormally spliced in AML compared to normal CD34^+^ HSC [35].

According to this global splicing deregulation in AML, we found increased levels of uPAR splicing variants in AML blasts as compared to normal CD34^+^ cells. Overexpression of the uPAR Δ5 variant (including its 3′UTR) promotes expression of pro-tumoral factors and significantly increases cell adhesion and migration, as previously shown for the ectopic expression of the sole 3′UTR of uPAR [24]. Expression of uPAR Δ5-3′UTR also moderately increases cell proliferation, unlike that of the 3′UTR. uPAR Δ5 effects on these cell activities should be attributed to the ceRNA activity of its transcript, since it is degraded so fast that the protein is not detected (Figure 3B). The unexpected effect of uPAR Δ5 expression on cell proliferation suggests that the 3′UTR of uPAR Δ5 might better expose new MREs as compared to isolated 3′UTR, thus recruiting additional miRs involved in cell proliferation.

The hypothesized role for previously identified and characterized uPAR variants was related to their translation in protein and not to the possible capability of these transcripts to regulate gene expression. In this context, it is interesting to point out that the mRNA of uPAR del 4/5 is a negative prognostic factor in breast cancer, while the encoded protein reduces invasion and metastasis in vivo [32]. We may speculate that, in breast cancer, the function of the alternatively spliced transcript may play a dominant pro-tumoral function independent of protein generation, that is the activity of molecular sponge for oncosuppressor miRNAs. This activity could promote the transcript degradation, rendering trifling its translation in a cancer-protective protein. Another interesting example in this context comes from CCR2, the receptor for the CCL2 chemokine. The CCR2 protein is implicated in cancer progression; however, higher *CCR2* mRNA level is associated with prolonged survival of breast cancer patients. The study of the non-coding function of *CCR2* mRNA shows that its 3′UTR inhibits breast cancer cell metastasis by repressing epithelial–mesenchymal transition (EMT) in vitro and suppresses breast cancer metastasis in vivo [20].

Our results suggest that, in leukaemia cells, uPAR may sustain a malignant phenotype not only at protein level but also at mRNA level.

In summary, we identified 3′UTR-containing variants of uPAR mRNA in AML cells and blasts; the most abundant variant, uPAR Δ5, was cloned and its overexpression in AML cells promoted expression of pro-tumoral factors and increase in biological activities, probably due to the ceRNA activity of its mRNA.

## 5. Conclusions

The importance of post-transcriptional regulation of gene expression in myeloid leukaemia has definitely emerged in the last decade. Dysregulation of splicing mechanisms, microRNA expression, RNA-methylation, ceRNA network and, recently, alternative polyadenylation, appear to play crucial roles in leukaemia development [14,18,35,36,37]. In this context, uPAR variants may represent a novel malignancy marker and their ceRNA activity should be taken into account in therapeutic strategies.

## Data Availability

The data presented in this study are available in this article and Supplementary Materials.

## Supplementary Materials

The following supporting information can be downloaded at: https://www.mdpi.com/article/10.3390/cancers14081980/s1, Original Western Blot Figures.
