# Supplementary figures and images for: Identification of uPAR Variants Acting as ceRNAs in Leukaemia Cells
Source: Cancers (Basel). 2022 Apr 14;14(8):1980. doi: 10.3390/cancers14081980 (PMC9025028; doi:10.3390/cancers14081980)

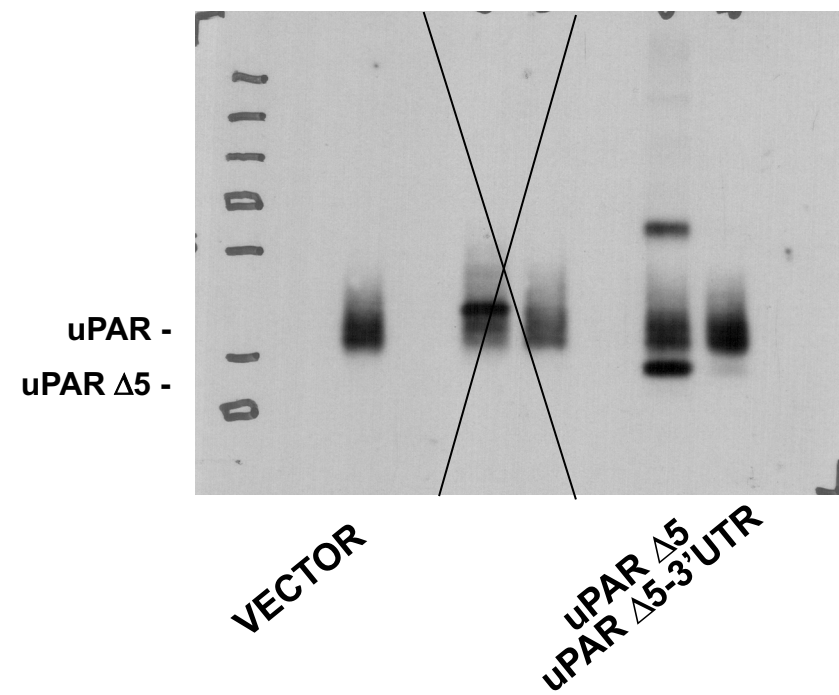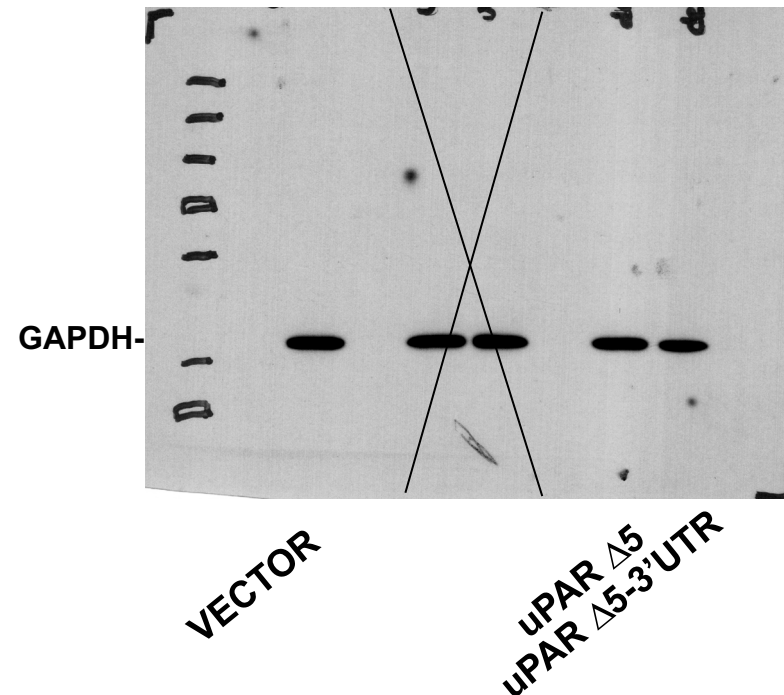

Figure S1. Original Western Blot of Figure 3.

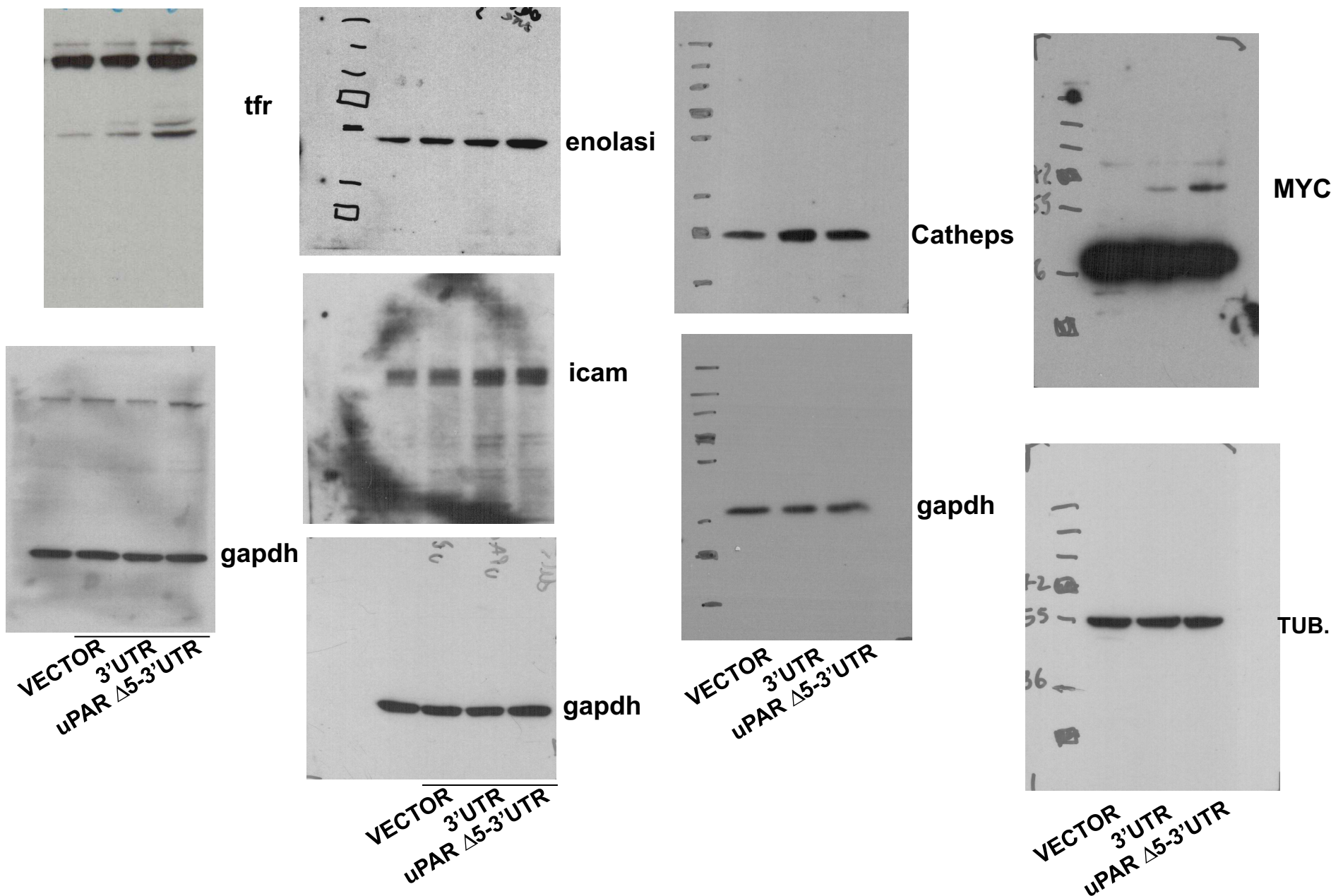

Figure S2. Original Western Blot of Figure 4A.

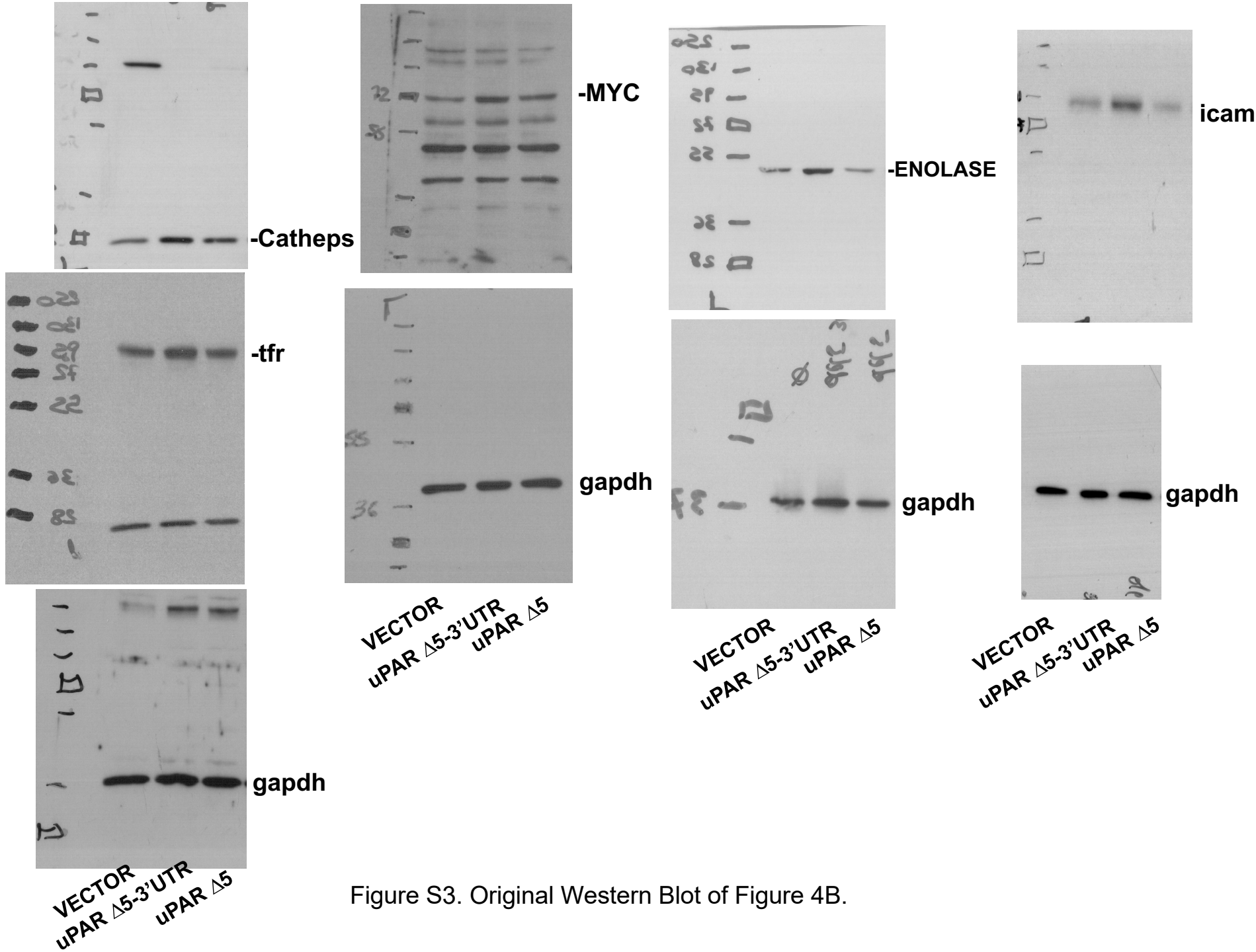

Supplement: Supplementary file 1 [file cancers-14-01980-s001.zip › cancers-1614897-supplementary.pdf]
